# Glycosidase Isoforms in Honey and the Honey Bee (*Apis mellifera* L.): Differentiating Bee- and Yeast-Derived Enzymes and Implications for Honey Authentication

**DOI:** 10.3390/insects16060622

**Published:** 2025-06-12

**Authors:** Ratko Pavlović, Sanja Stojanović, Marija Pavlović, Nenad Drulović, Miroslava Vujčić, Biljana Dojnov, Zoran Vujčić

**Affiliations:** 1 Department of Biochemistry, Faculty of Chemistry, University of Belgrade, Studentski trg 12-16, 11000 Belgrade, Serbia; zvujcic@chem.bg.ac.rs; 2 Department of Chemistry, Institute of Chemistry, Technology and Metallurgy, University of Belgrade, Njegoševa 12, 11000 Belgrade, Serbia; sanja.stojanovic@ihtm.bg.ac.rs (S.S.); marija.pavlovic@ihtm.bg.ac.rs (M.P.); nenad.drulovic@ihtm.bg.ac.rs (N.D.); miroslava.vujcic@ihtm.bg.ac.rs (M.V.); biljana.dojnov@ihtm.bg.ac.rs (B.D.)

**Keywords:** transfructosidase, transglucosidase, α-glucosidase, β-fructofuranosidase, isoelectric point

## Abstract

The enzymes in honey can originate not only from bees and the plants from which the bees collect pollen and nectar but also from feed provided by beekeepers. Enzymes that hydrolyze sucrose—present in honey (α-glucosidase) or honey adulterated with invert syrup (β-fructofuranosidase)—can be distinguished using zymography, where enzymatic bands are detected with nitroblue tetrazolium (NBT) after sugar removal via ultrafiltration. This method enables the identification of honey produced in hives that have been improperly fed with invert syrup, leading to the mixture of natural honey and syrup, and offers a practical tool to detect indirect adulteration. The NBT assay, in combination with ultrafiltration, was used to determine the isoelectric point of honey bee α-glucosidases. The pI value of 6.63 for isoforms found in the head, midgut, and natural honey extracts during winter can be attributed to α-glucosidase III. Two additional isoforms with isoelectric points of 5.20 and 5.77 were observed in the midgut extract and may correspond to α-glucosidase I and II. The difference between α-glucosidase and β-fructofuranosidase was confirmed using a substrate specificity test, followed by thin-layer chromatography, where it was confirmed that α-glucosidase from natural honey, bee head, and bee midgut does not hydrolyze raffinose, in contrast to yeast β-fructofuranosidase.

## Simple Summary

In an effort to increase honey yields for commercial purposes, beekeepers may feed colonies with sugar-based supplements, such as invert syrup. These supplements can introduce enzymes of non-bee origin into the hive. In this study, we partially purified and characterized honey bee α-glucosidase, which is responsible for hydrolyzing sucrose in nectar. We compared it to β-fructofuranosidase, an enzyme that is present in commercial invert syrup, exhibits similar activity, and originates from brewer’s yeast. Despite catalyzing the same reaction, these enzymes showed distinct electrophoretic mobility in native polyacrylamide gels and could be clearly differentiated using NBT (nitroblue tetrazolium) zymography. Furthermore, we determined the isoelectric point (pI) of bee-derived α-glucosidase using this method. To our knowledge, this is the first report of its kind. These findings provide a useful tool for identifying the origin of enzymes present in honey and for assessing the impact of artificial feeding on honey authenticity. Our results highlight the importance of distinguishing between bee- and non-bee-derived enzymes in honey quality control and contribute to a better understanding of the biochemical characteristics of bee enzymes involved in carbohydrate metabolism.

## 1. Introduction

Poor nutrition is the leading cause of bee colony deaths [1], and high-quality and abundant nutrition is necessary for the development of individual bees and complete bee colonies [2]. Malnutrition can lead to increased susceptibility to pesticides and diseases [3,4], and an imbalanced diet can negatively affect a honey bee colony [5]. Colonies lacking essential nutrients produce undernourished, short-lived workers, which weakens the colony and its ability to resist stressors [6,7]. During periods when bee forage is scarce or after honey has been harvested, beekeepers often replace the natural source of carbohydrates (nectar) collected by the bees with different carbohydrates, such as sucrose, invert syrup, or high-fructose corn syrup [8,9]. Sucrose syrup derived from sugar beet or sugar cane has been proven to be an effective carbohydrate substitute in honey bee diets [10,11]. When such syrups are provided by beekeepers as a replacement for natural nectar, bees incorporate them into their food stores and continuously reorganize them to maintain the typical concentric pattern of brood, pollen, and honey on the comb, relocating them during both the spring expansion and the autumn preparation for winter [12]. However, these artificial carbohydrate sources differ from natural nectar in both the sugar composition and the origin and properties of the enzymes [13], and their use may impact honey composition and authenticity, especially if beekeepers do not follow good beekeeping practices (apitechnical measures) to prevent supplemented syrup from entering the honey intended for human consumption.

An equimolar mixture of glucose and fructose, known as invert syrup, is widely used in the food industry and to produce artificial honey [14]. Baker’s yeast, *Saccharomyces cerevisiae*, is the most significant source of the enzyme invertase (β-fructofuranosidase, EC 3.2.1.26) [15], which is the dominant enzyme in the production of invert syrup used to feed honey bees. Historically termed “invertase,” this honey bee enzyme responsible for sucrose hydrolysis is now classified as α-glucosidase (EC 3.2.1.20), distinct from yeast β-fructofuranosidase (EC 3.2.1.26), which is the enzyme currently recognized as invertase in biochemical nomenclature (BRENDA; Expasy). This distinction is important, as both enzymes fall under the glycosidase class (EC 3.2.1.-), which catalyzes the hydrolysis of glycosidic bonds, yet they differ in substrate specificity and biological origin. Thick invert syrup resists crystallization better than sucrose syrup, which is a desirable property for commercial beekeepers [13]. Recently, invert syrup has been considered as a substitute with the potential to further improve the health of a bee colony, because the bees are not forced to produce enzymes to break down sucrose, unlike when they are fed sucrose syrup [10]. In our previous work, we demonstrated that bees add less amylase into invert syrup than into sucrose syrup [13], which aligns with previous findings that higher amylase activity was observed in honey from colonies supplemented with sucrose syrup compared to those fed invert sugar syrup [16]. However, any supplemental feeding of honey bees carries the risk that components of the feed provided—regardless of origin—may end up in the honey intended for human consumption.

Recently, we demonstrated that bees process sucrose and invert syrup differently [13], resulting in differences in the sugar composition of the honey produced, particularly in the oligosaccharide profile. These differences are likely influenced by the composition of the feeding syrups and the enzymatic activity expressed by the bees in response to different dietary inputs [17,18,19]. The yeast enzyme β-fructofuranosidase catalyzes transfructosylation to produce fructo-oligosaccharides, such as kestose, nystose, and fructofuranosyl nystose [20]. In contrast, honey bee glycosidase catalyzes the transglycosylation reaction to form various α-glucosylated compounds, including oligosaccharides [18,19], and a major product from sucrose, erlose [21]. Non-enzymatic transglycosylation reactions also contribute to the oligosaccharide diversity in honey (maltose, isomaltose, inulobiose, sophorose, gentiobiose, 1-kestose, and panose) [22].

The composition of honey, particularly the proportion of different sugars, is highly complex and influenced by various factors [18,19,22]. The presence of specific enzymes can serve as a valuable indicator of honey origin. While heat treatment may be used to inactivate foreign enzyme activity, it also increases HMF concentration [23], alters color, and degrades naturally occurring enzymes in honey [24].

Honey bees have several α-glucosidases that hydrolyze sucrose [25], typically the main carbohydrate in nectar. Other sugars in nectar occur in small amounts compared to the three dominant sugars [26], including monosaccharides, (e.g., mannose, arabinose, xylose), disaccharides (maltose, melibiose) or, more rarely, oligosaccharides (raffinose, melezitose, stachyose). All are derived from sucrose translocated in phloem sap or synthesized in the nectary [27]. Bees use the resulting glucose and fructose as an energy source or to produce honey [28]. The main honey sugars are fructose (32–44%) and glucose (23–38%) [29].

The enzyme α-glucosidase (EC 3.2.1.20) is the most abundant protein—accounting for about 50% of the total proteins—in the hypopharyngeal gland of bees, with a mass of 70 kDa [30] and an optimum pH of 5.0 [31]. α-Glucosidase I was isolated and characterized from *Apis cerana*; it has a molecular mass of 82 kDa, an optimum pH of 5.0, and is stable up to 40 °C [32]. The characterization of α-glucosidase is essential for understanding carbohydrate metabolism in honey bee colonies. Although this enzyme has been extensively studied and is also present in honey, its properties have not yet been fully elucidated. The isoelectric point of honey bee α-glucosidase has not been published until now. Another aim of our research was to investigate the potential for detecting honey adulteration by comparing enzyme profiles between honey bee α-glucosidase and β-fructofuranosidase from yeast, with the latter being commonly used in the production of invert syrup [15], which is frequently associated with honey adulteration [33].

## 2. Materials and Methods

### 2.1. Reagents

All reagents and solvents used in this study were of the highest available purity and were purchased from Merck, Sigma-Aldrich, Qiagen, Hilden, Germany. A sample of natural polyfloral honey was obtained from honey extracted from an urban apiary in Vršac, Serbia, with various flowering plant species present in the surrounding area, where the most dominant honey-bearing plants include black locusts (*Robinia pseudoacacia*), lindens (*Tilia* spp.), and sunflowers (*Helianthus annuus*).

### 2.2. Honey Bee Collection, Dissection, and Sample Preparation

**Figure 1:**
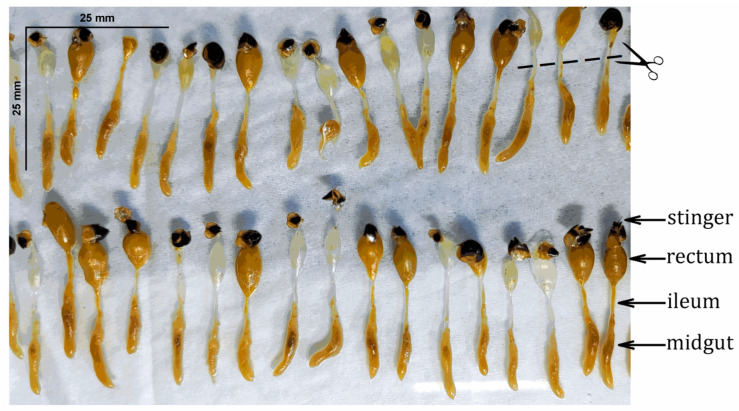
Groups of 50 honey bees were dissected simultaneously.

In February (winter bees), we collected 100 forager bees from the landing board of five different honey bee colonies. Winter bees were selected because their midguts retain α-glucosidase III, allowing for a comparative analysis across tissues [30]. The gut (midgut and rectum) was rapidly removed using tweezers after immobilizing the bees in a refrigerator [34]. The midgut and rectum were removed together with the stinger. The midgut was divided from the rectum, in the middle of the ileum, using a surgical scalpel. One half of the ileum remained with the midgut, while the other half remained with the rectum, as described in [35]. The midguts were separated on a cold glass plate, and the heads were collected to form pooled samples. For α-glucosidase characterization, groups of 50 honey bees—after being immobilized in a refrigerator at 4 °C—were dissected on ice simultaneously (Figure 1).

Following homogenization and extraction as described previously [36], the samples were then concentrated via ultrafiltration using Amicon^®^ Ultra-15 Centrifugal Filter Unit (10 kDa, Merck Millipore, Burlington, MA, USA). Molecules smaller than 10 kDa were removed from all the samples by periodic buffer exchange, as the reducing sugars present in honey would react with NBT, making the zymographic detection of glycosidases impossible. Size exclusion chromatography was performed on an FPLC system using a Sephacryl S-300 HR matrix (Cytiva, Uppsala, Sweden). The column volume was 300 mL, and it was equilibrated with 20 mM acetate buffer (pH 5.5). The samples (5 mL each) were applied, and elution was carried out with the same buffer at a flow rate of 1.5 mL/min. Fractions of 5 mL were collected. After chromatography, glycosidase activity in the obtained fractions was determined using the DNS assay [37], as previously described [13]. The fractions exhibiting the highest α-glucosidase activity (MG19, HE21 and HO18) were selected for further analyses.

### 2.3. Isoelectric Focusing and Zymogram Detection of Glycosidase Activity

Glycosidase isoforms in selected ultraflitrated extracts and fractions after size exclusion chromatography were detected through the zymogram method, using in-gel activity staining following electrophoretic separation after Native PAGE [38] or isoelectric focusing electrophoresis (IEF). Native PAGE was performed using a vertical electrophoresis unit (Hoefer SE260, Hoefer Inc., Holliston, MA, USA). Gels (30% T, 2.7% C) were cast with 1.5 M Tris-HCl buffer (pH 8.8) for the separating gel and 0.5 M Tris-HCl (pH 6.8) for the stacking gel. Electrophoresis was run at 80 V until the samples entered the separating gel, then continued at 125 V. The running buffer was 0.025 M Tris, 0.192 M glycine, pH 8.3. IEF were performed using a hand-cast 7 cm acrylamide gel (7.5%) with a linear pH gradient ranging from 3.5 to 9.5, prepared with co-polymerized ampholytes (Amersham Biosciences, Uppsala, Sweden). The gel solution consisted of 3.75 mL of 30% acrylamide, 0.75 mL of ampholytes (pI 3.5–9.5), 4.0 mL of 50% glycerol, 6.5 mL of distilled water, 75 μL of 10% ammonium persulfate (APS), and 12 μL of TEMED. The solution was degassed prior to polymerization. Isoelectric focusing was performed according to the manufacturer’s instructions (Multiphor II, LKB Pharmacia, Uppsala, Sweden). The run was carried out for 1 h and 50 min at 1050 V, 8.0 mA, with a constant power of 7 W, at 10 °C.

After Native PAGE, the gel was incubated in a 5% sucrose solution dissolved in 50 mM sodium acetate buffer, pH 6.0, at 35 °C for 60 min. After IEF, the gel was incubated in another 5% sucrose solution dissolved in 50 mM sodium acetate buffer, pH 5, at 35 °C for 30 min. The reaction was terminated by the addition of 100 mL of 0.3 M NaOH solution. Reducing sugars were detected by the NBT (Nitro blue tetrazolium chloride) solution dissolved in 0.3 M NaOH to a final concentration of 1 mg/mL [39]. The gel was handled as carefully and gently as possible, with minimal shaking, primarily because the products of sucrose hydrolysis (glucose and fructose) are highly mobile in the gel. This can easily lead to the spreading of the stain formed by the insoluble dye complex resulting from the reaction between NBT and the reducing sugars, the products of sucrose hydrolysis.

### 2.4. Determination of α-Glucosidase pI Values

During the sample application onto the IEF gel, one well was left empty to allow for pI gradient determination. After completing the IEF run, the corresponding gel segment without sample was removed and cut into pieces measuring 0.5 × 0.7 mm. The gel pieces were placed in separate vials containing 7 mL of distilled water that had been boiled and cooled to remove CO_2_. The gel was then shaken for 90 min in distilled water, after which the pH of each vial was measured. Based on the measured pH values and corresponding gel positions, a graph was plotted to determine the pI value (y = 0.0646x + 3.2021, R^2^ = 0.9863), with the band positions previously determined using ImageJ 1.54g software (NIH, Bethesda, MD, USA).

### 2.5. Analysis of Sugar Hydrolysis Products

To confirm the difference in substrate specificity between α-glucosidase and β-fructofuranosidase, 15 μL of a 10-fold diluted sample (ultrafiltrated and concentrated extracts from the head, midgut, and honey, and the purified β-fructofuranosidase isolated from *Saccharomyces cerevisiae*) was mixed with 135 μL of raffinose (1 mg/mL). The reaction was carried out for 30 min at 35 °C and stopped by boiling for 3 min. The resulting samples were applied to a thin-layer chromatography (TLC) plate measuring 4.5 cm × 6 cm (Silica gel 60 F254, Merck, Darmstadt, Germany).

Approximately 1 μL of each sample and the standard sugars was applied. The mobile phase consisted of n-butanol/ethanol/water/acetic acid (5:3:2:0.5; *v*/*v*/*v*/*v*). The analysis was performed at room temperature (22 ± 2 °C). For sugar visualization, the plates were treated with an α-naphthol solution (0.5 g α-naphthol dissolved in 95 mL of 96% ethanol and 5 mL of concentrated H_2_SO_4_), followed by heating at 150 °C for 5 min.

## 3. Results

### 3.1. Characterization of Honey Bee α-Glucosidases

**Figure 2:**
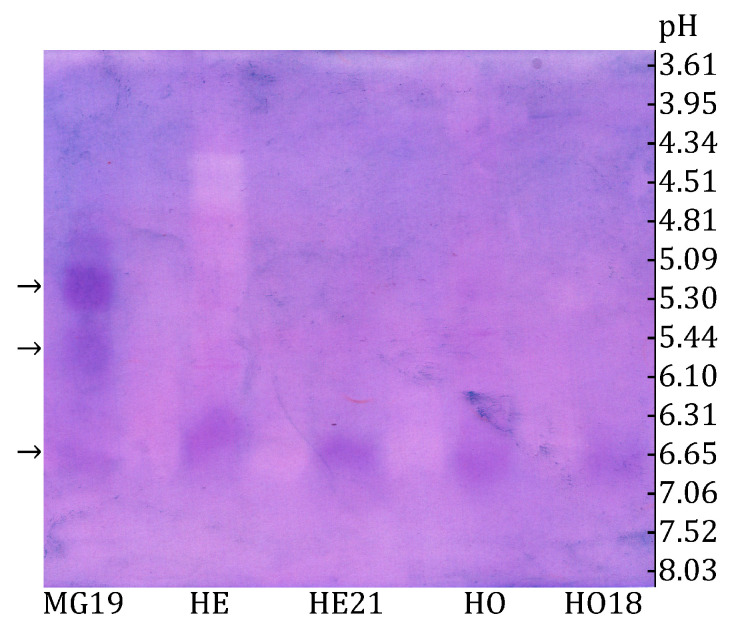
Zymogram detection of honey bee α-glucosidases after IEF using sucrose and NBT reagent in ultrafiltrated extracts of honey bee head (HE) and honey (HO), as well as in selected fractions obtained after size exclusion chromatography: MG19—midgut fraction, HE21—head fraction, and HO18—honey fraction. Isoforms are indicated by arrows (left) and the marked pH values measured in the gel after IEF (right).

The three size exclusion chromatography fractions exhibiting the highest α-glucosidase activity were analyzed using the zymogram method. As shown in Figure 2, the zymogram after IEF revealed at least three distinct forms of α-glucosidase in the midgut extract fraction (MG 19), as well as one α-glucosidase band appearing at the same position in all other samples (HE, HE21, HO, and HO18).

**Figure 3:**
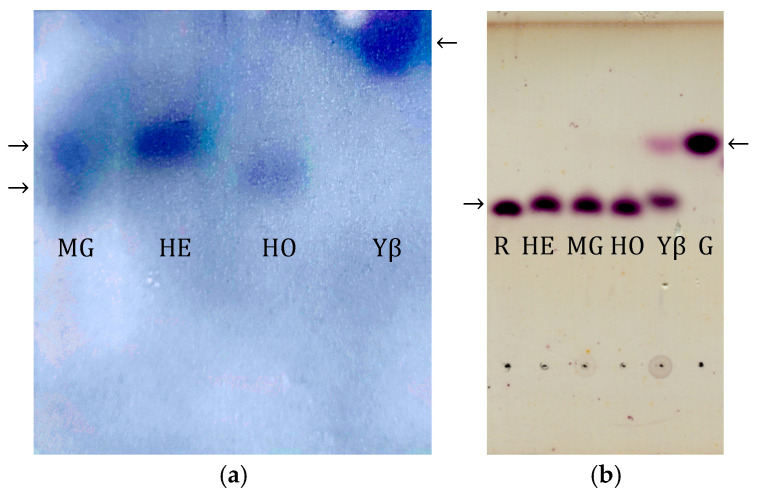
(**a**) Zymogram detection of honey bee α-glucosidases from midgut (MG), head (HE), and honey (HO) and yeast β-fructofuranosidase (Yβ) after Native PAGE; isoforms are indicated by arrows, note slower migration of yeast β-fructofuranosidase (Yβ) (**b**) TLC analysis of saccharides obtained after enzymatic assay with raffinose. Bee enzymes (HE, MG, HO) did not hydrolyze raffinose, confirming their α-glucosidase identity, unlike yeast β-fructofuranosidase (Yβ). Standards, raffinose (R) and glucose (G), are designated by arrows.

As shown in Figure 3a, the zymogram obtained after Native PAGE demonstrates that honey bee α-glucosidases extracted from the midgut (MG), head (HE), and honey (HO) exhibit faster migration compared to yeast β-fructofuranosidase. These results suggest that honey bee α-glucosidases have a lower molecular mass in comparison to yeast β-fructofuranosidase, indicating structural and possibly functional differences between the two types of enzymes.

### 3.2. Distinguishing Between α-Glucosidase and β-Fructofuranosidase

To investigate the substrate specificity of the detected glycosidases, which determines the enzyme type (i.e., whether the present enzyme is an α-glucosidase or a β-fructofuranosidase), an assay with raffinose was performed. The resulting products were separated using thin-layer chromatography (TLC) and detected with an α-naphthol solution (Figure 3b). The obtained results revealed that ultrafiltrated honey bee α-glucosidase extracted from the head (HE), midgut (MG) and honey (HO) do not hydrolyze raffinose. On the contrary, purified β-fructofuranosidase isolated from *Saccharomyces cerevisiae* (Yβ) hydrolyzes raffinose.

## 4. Discussion

We collected bees at the hive entrance because forager bees are the most abundant at this location and exhibit significantly higher α-glucosidase activity compared to younger bees [30]. Immunological techniques confirmed that α-glucosidase I is present in the midgut, α-glucosidase II is found in both the midgut and hemolymph, and α-glucosidase III is located in the hypopharyngeal gland, from where it is secreted into the nectar collected by bees [40]. As a result, α-glucosidase III is the enzyme present in honey [40]. It is also detected in the midgut during winter [40], which is why we chose to collect winter bees.

Although honey bee α-glucosidases have been partially characterized [30,31,32], their isoelectric point has not been previously published. The isoelectric point is the pH value at which a protein has a net zero charge [41]. This is important because pH influences protein solubility, stability, and interactions with other molecules [41].

The band visible in all samples is most likely α-glucosidase III, as this is the only honey bee α-glucosidase present in the head (specifically in the hypopharyngeal glands), honey, and midgut in the winter [40]. The bees used in this experiment were collected from the hive entrance on 23 February, when only winter bees were present inside the colonies. This explains why a band at the same position as in the head and honey is also observed in the midgut. The determined isoelectric point of α-glucosidase III was 6.63 (Figure 2).

Besides α-glucosidase III, two additional bands were observed in fraction 19 (MG19) of the midgut extract (Figure 2). One had an isoelectric point of 5.20, while the other had a pI of 5.77. These may correspond to α-glucosidase I and II, as immunological evidence has confirmed that two α-glucosidases (designated as α-glucosidase I and II) are distinct from α-glucosidase III and are present in the honey bee midgut [40]. The findings presented in Figure 2 confirm that α-glucosidase III is secreted in the head, then transferred to the midgut or added to the honey by the bees. To our knowledge, this is the first report of the pI of honey bee α-glucosidase III, a critical parameter for enzyme purification and identification.

It is known that some invert syrups show high residual enzymatic activity [10]. The zymogram following native electrophoresis (Figure 3a) allows for the visual distinction of bands originating from α-glucosidase and β-fructofuranosidase, due to their different electrophoretic mobilities and substrate specificities. This method can be applied for detecting honey adulteration, as the presence of β-fructofuranosidase—which is not naturally found in honey—suggests the possible addition of invert syrups or other sweeteners. We found that yeast-derived β-fructofuranosidase, typically used for invert syrup preparation, retains its activity both in the honey stored by bees in the comb and in the bee midgut [13]. The presence of both bee- and yeast-derived glycosidases suggests unintentional contamination from supplemental feeding, whereas the exclusive presence of yeast enzymes points to intentional adulteration. Similar techniques have been used to detect foreign amylases in honey, supporting the effectiveness of Native PAGE in identifying enzymatic additives that could indicate honey adulteration [42].

The results presented in Figure 3b show that the enzymes found in the midgut and head of the honey bee, as well as those found in natural honey, do not hydrolyze raffinose, which is in line with the results previously published by Kimura et al., 1990 [43]. This confirms that the honey bee enzyme responsible for sucrose hydrolysis is α-glucosidase [40]. In contrast, purified β-fructofuranosidase isolated from *Saccharomyces cerevisiae* (Yβ) hydrolyzes raffinose, in line with the results published by Liu et al., 2021 [44].

## 5. Conclusions

We determined the isoelectric point of α-glucosidase III and gained new insights into the distribution of α-glucosidases in different tissues and honey. Using zymography after IEF allowed for the detection of at least three α-glucosidase isoforms, with α-glucosidase III consistently present in all the samples tested, including honey, head, and midgut extracts. The results confirm that α-glucosidase III, secreted from the hypopharyngeal glands, is the predominant enzyme in honey and remains present in the midgut during winter.

Moreover, zymograms following native electrophoresis proved to be a simple and effective method for distinguishing ultrafiltration-concentrated enzymes in natural or adulterated honey, specifically α-glucosidase and β-fructofuranosidase. Zymographic detection has been recognized for its high sensitivity [45,46]. In the future, this approach has the potential to differentiate other enzymes that may be present in honey adulterants. This could serve as a valuable technique in quality control and authenticity testing of honey by identifying the presence of non-native enzymes linked to adulteration. Future research should explore the application of this method in routine honey authentication protocols.

## Data Availability

The original contributions presented in this study are included in the article. Further inquiries can be directed to the corresponding author.
